# An Account of the Spontaneous Discharge of Water through the Navel, in a Case of Dropsy

**Published:** 1799-11

**Authors:** John Pearson

**Affiliations:** senior Surgeon *of the* Lock Hospital *and* Asylum, *and of the* Public Dispensary; *Reader on the Principles and Practice of Surgery*


### *An Account of the Spontaneous Difcharge of Water through the Navel, in a Cafe of Dropfy, by* JOHN PEARSON, fenior Surgeon *of the* Lock Hofpital *and* Afylum, *and of the* Public Difpenfary; *Reader on the Principles and Practice of Surgery.*

IN the month of June 1783, I was defired to vifit Mrs. H. who was afflicted with a dropfy; fhe was forty-five years of age; the catamenia had ceafed to appear during a few months, and fhe had lately become very corpulent. The operation of the paracentefis was the only probable mean of relieving her; but fhe was extremely averfe to it, and continued to take various medicines till the month of September, when being much increafed in fize, and fuffering great uneafinefs, fhe confented to undergo the operation.

On examining the abdomen, I found on the left fide, a large, hard, moveable tumour, which determined me to puncture the right fide, and I drew off eight quarts of a greenifh coloured water. She took the the ufual medicines, to prevent if poffible a fecond collecting of the fluid; the remedies however proved ineffectnal, and it became neceffary to repeat the operation, at the latter end of December. About two gallons of a greenifh water were again difcharged; the tumour was much larger than in September, and it gave her fo much trouble, that fhe found it very inconvenient either to walk or to fit.

Mrs. H. being anxious to avoid a repetition of the operation, by the advice of a female friend, applied a plafter of frankincenfe, large enough to cover the greater part of the abdomen. In the courfe of a few days, the navel acquired an unufual fenfibility, and water was perpetually oozing from under the plafter; fo that it became neceffary for her to wear a fheet folded feveral times, in order to abforb it. The plafter was renewed once a week, and the water continued to flow through the navel; the difcharge was not conftant, but returned every five or fix days, and from one to two pints was evacuated each day, during two, and fometimes three days fucceffively. She complained that thefe periodical difcharges of water produced great languor and feeblenefs; but they evidently prevented the accumulation of fluid in the cavity of the peritonæum. On examining the umbilicus, I found it very red, large, and prominent, refembling a rafpberry on its external furface; and a great number of minute orifices could be diftinguifhed, through which the water proceeded, as from a fponge. Mrs. H. was regularly regularly relieved by this periodical evacuation of the water, from the latter end of December 1783, to the middle of March 1784; and during this period, ſhe gained no addition to her ſize; but having become weary of the attention which this ſtate of her diſeaſe required, by the advice of her apothecary, ſhe diſcontinued the plaſter, and waſhed the navel with port wine, in which a quantity of oak bark had been infuſed. This lotion reſtrained the diſcharge of water completely, nor could it ever be reſtored afterwards by the repeated application of the frankincenſe. From this period the water began to diſtend the abdomen, and on May the 11th I repeated the operation, and diſcharged twelve pints of a coffee coloured fluid, with a large baſon full of jelly-like ſubſtance, mixed with pus. Her ſtrength was at this time greatly reduced, and ſhe only lived to the beginning of June. I was not permitted to open the body. The extraordinary effects which followed the application of frankincenſe in this inſtance, induced me to adviſe a trial of it in ſeveral caſes of the aſcites; but I have never ſeen the leaſt advantage derived from the plaſter to any patient ſince that time.

GOLDEN SQUARE, *September* 17, 1799.

